# Effects of the CYP3A4*1B Genetic Polymorphism on the Pharmacokinetics of Tacrolimus in Adult Renal Transplant Recipients: A Meta-Analysis

**DOI:** 10.1371/journal.pone.0127995

**Published:** 2015-06-03

**Authors:** Wei-Long Shi, Hui-Lin Tang, Suo-Di Zhai

**Affiliations:** Department of Pharmacy, Peking University Third Hospital, Beijing, China; University of Toledo, UNITED STATES

## Abstract

**Background and Objective:**

The association between the CYP3A4*1B single nucleotide polymorphism (SNP) and tacrolimus pharmacokinetics in different studies is controversial. Therefore, a meta-analysis was employed to evaluate the correlation between the CYP3A4*1B genetic polymorphism and tacrolimus pharmacokinetics at different post-transplantation times in adult renal transplant recipients.

**Methods:**

Studies evaluating the CYP3A4*1B genetic polymorphism and tacrolimus pharmacokinetics were retrieved through a systematical search of Embase, PubMed, the Cochrane Library, ClinicalTrials.gov and three Chinese literature databases (up to Sept. 2014). The pharmacokinetic parameters (weight-adjusted tacrolimus daily dose and tacrolimus trough concentration/weight-adjusted tacrolimus daily dose ratio) were extracted, and the meta-analysis was performed using Stata 12.1.

**Results:**

Seven studies (involving 1182 adult renal transplant recipients) were included in this meta-analysis. For the weight-adjusted tacrolimus daily dose, in all included renal transplant recipients (European & Indian populations), CYP3A4*1/*1 recipients required a significantly lower weight-adjusted tacrolimus daily dose than did CYP3A4*1B carriers at 7 days (WMD -0.048; 95% CI -0.083 ~ -0.014), 6 months (WMD -0.058; 95% CI -0.081 ~ -0.036) and 12 months (WMD - 0.061; 95% CI -0.096 ~ -0.027) post-transplantation. In light of the heterogeneity, the analysis was repeated after removing the only study in an Indian population, and CYP3A4*1/*1 European recipients (mostly Caucasian) required a lower weight-adjusted tacrolimus daily dose within the first year post-transplantation. The tacrolimus trough concentration/weight-adjusted tacrolimus daily dose ratio (C_0_/Dose ratio) was significantly higher in CYP3A4*1/*1 recipients than in CYP3A4*1B carriers at 6 months (WMD 52.588; 95% CI 22.387 ~ 82.789) and 12 months (WMD 62.219; 95% CI 14.218 ~ 110.221) post-transplantation. When the only study in an Indian population was removed to examine European recipients (mostly Caucasian), the significant difference persisted at 1 month, 6 months and 12 months post-transplantation.

**Conclusion:**

Based on our meta-analysis, the CYP3A4*1B genetic polymorphism affects tacrolimus dose requirements and tacrolimus trough concentration/weight-adjusted tacrolimus daily dose ratio within the first year post-transplantation in adult renal transplant recipients, especially in European recipients (mostly Caucasian).

## Introduction

Renal transplantation is an effective treatment for the patients with end-stage renal disease. Tacrolimus, a macrolide antibiotic compound, is the most frequently used maintenance immunosuppressant after renal transplantation [1]. However, tacrolimus is characterized by its narrow therapeutic index and significant inter-individual variability in pharmacokinetics. Tacrolimus blood concentration below target trough levels can lead to rejection, and higher trough blood concentrations can lead to toxicity and infection [2,3]. Achieving a steady target blood concentration is critical to avoid rejection and adverse drug effects [4]. However, several factors influence the pharmacokinetics of tacrolimus, including hepatic dysfunction, post-transplantation time, hematocrit, serum albumin, age, race and drug interactions, especially gene polymorphism [5]. Single nucleotide polymorphisms (SNPs) in *cytochrome P450 3A* (*CYP3A*) play an important role in tacrolimus metabolism [6]. CYP3A enzymes in human liver microsomes play a major role in the oxidation of tacrolimus[7], and the tacrolimus metabolism within the small intestinal contributes significantly to its bioavailability[8,9]. Many studies in renal transplant recipients focus on CYP3A5*3 genetic polymorphism (rs776746, 6986A>G). There is a widespread view that *CYP3A5* nonexpressers (CYP3A5*3/*3 carriers) required lower mean tacrolimus doses [10] and exhibit higher trough concentration/dose ratios [11,12]. The CYP3A4*1B genetic polymorphism (rs2740574, −392A>G), linked to enhanced CYP3A4 activity, is likely related to the rapid metabolism of tacrolimus [6], but the effect of the CYP3A4*1B genetic polymorphism on tacrolimus pharmacokinetics (dose and concentration) in renal transplant recipients is controversial [13], and there has been no meta-analysis to assess the issue to date.

To evaluate the correlation between the CYP3A4*1B genetic polymorphism and tacrolimus pharmacokinetics (weight-adjusted tacrolimus daily dose and tacrolimus trough concentration/weight-adjusted tacrolimus daily dose ratio), a meta-analysis was employed to systematically review the published evidence of the relationship between the CYP3A4*1B genetic polymorphism and tacrolimus pharmacokinetics in adult renal transplant recipients.

## Methods

### Search strategy and study selection

Embase, PubMed, the Cochrane Library, ClinicalTrials.gov and three Chinese databases (CNKI, Sinomed and WanFang Data) were searched from their date of inception to September 2014, without language and publication status restrictions, for published studies that evaluated the effects of the CYP3A4*1B genetic polymorphism on tacrolimus pharmacokinetics. The search terms ((“tacrolimus” or “FK506”) and “CYP3A4”) as well as related Chinese keywords in the Chinese databases were used. In addition, the reference lists of the included articles and relevant reviews were searched manually. In cases of missing data, the original authors were contacted for more detailed information by e-mail.

The inclusion criteria for the included studies were as follows: (a) studies focus on the effects of the CYP3A4*1B genetic polymorphism on adult renal transplant recipients treated with tacrolimus; (b) tacrolimus pharmacokinetics parameters was described separately according to different CYP3A4*1B genotypes; and (c) tacrolimus pharmacokinetic parameters were measured at explicit post-transplantation times. According to the above criteria, studies were assessed independently by two reviewers (S.W.L. and T.H.L.) for inclusion in the meta-analysis.

### Data extraction and quality assessment

Relevant data from all eligible studies were extracted independently by the two reviewers (S.W.L. and T.H.L.), and discrepancies in the data extraction were resolved through consensus. The following information was collected: first author, publication information, design of the study, demographic data, immunosuppressive protocol, method of concentration measured, genotype frequency, post-transplantation time, weight-adjusted tacrolimus daily dose (Dose), tacrolimus trough concentration (C_0_), C_0_/Dose ratio. For continuous data, information was collected as mean (SD), if the studies provided the median (range), the method reported by Hozo *et al*.[14] was employed to estimate the mean (SD).

The quality of the included studies was assessed by two reviewers (S.W.L. and T.H.L.) through a checklist derived from the Strengthening the Reporting of Genetic Association (STREGA) recommendations for reports on genetic association studies [15], and modified according to the quality checklist described elsewhere[16,17].

### Statistical analysis

The Dose and C_0_/Dose ratio values were compared between CYP3A4*1/*1 recipients and CYP3A4*1B carriers, and a random-effect model was used for all meta-analyses. The data of the CYP3A4*1B carriers were calculated from the CYP3A4*1/*1B and the CYP3A4*1B/*1B groups using the method provided by Table 7.7.a of the Cochrane handbook 5.1.0 [18]. The continuous data were pooled by weighted mean difference (WMD) or standard mean difference (SMD) and 95% confidence interval (CI), and Z-tests were performed to determine the statistical significance of the results. Statistical significance was defined as *P* < 0.05.

The heterogeneity across the included studies was assessed using the *I* ^2^ statistic, with significance defined as *I* ^2^ > 50%. In case of substantial heterogeneity (*I* ^2^ > 50%), meta-regression was performed to explore the sources of heterogeneity [post-transplantation time (7 days, 1 month, 3 months, 6 months, 12 months), ethnicity (Caucasian, Indian, mixed race), location (Europe, India), method of concentration measured (MEIA, CMIA, EMIT), initial dose (0.1–0.16 mg/kg/day, 0.2–0.3 mg/kg/day), and Hardy-Weinberg equilibrium (equilibrium or disequilibrium)]. Further subgroup analysis was performed according to the results of the meta-regression. A sensitivity analysis was performed to assess the validity of the outcomes by excluding each observation successively. A publication bias analysis was not performed because less than 10 studies were included. All statistical analysis was performed using Stata 12.1.

## Results

### Characteristics of the articles included in the meta-analysis

**Fig 1:**
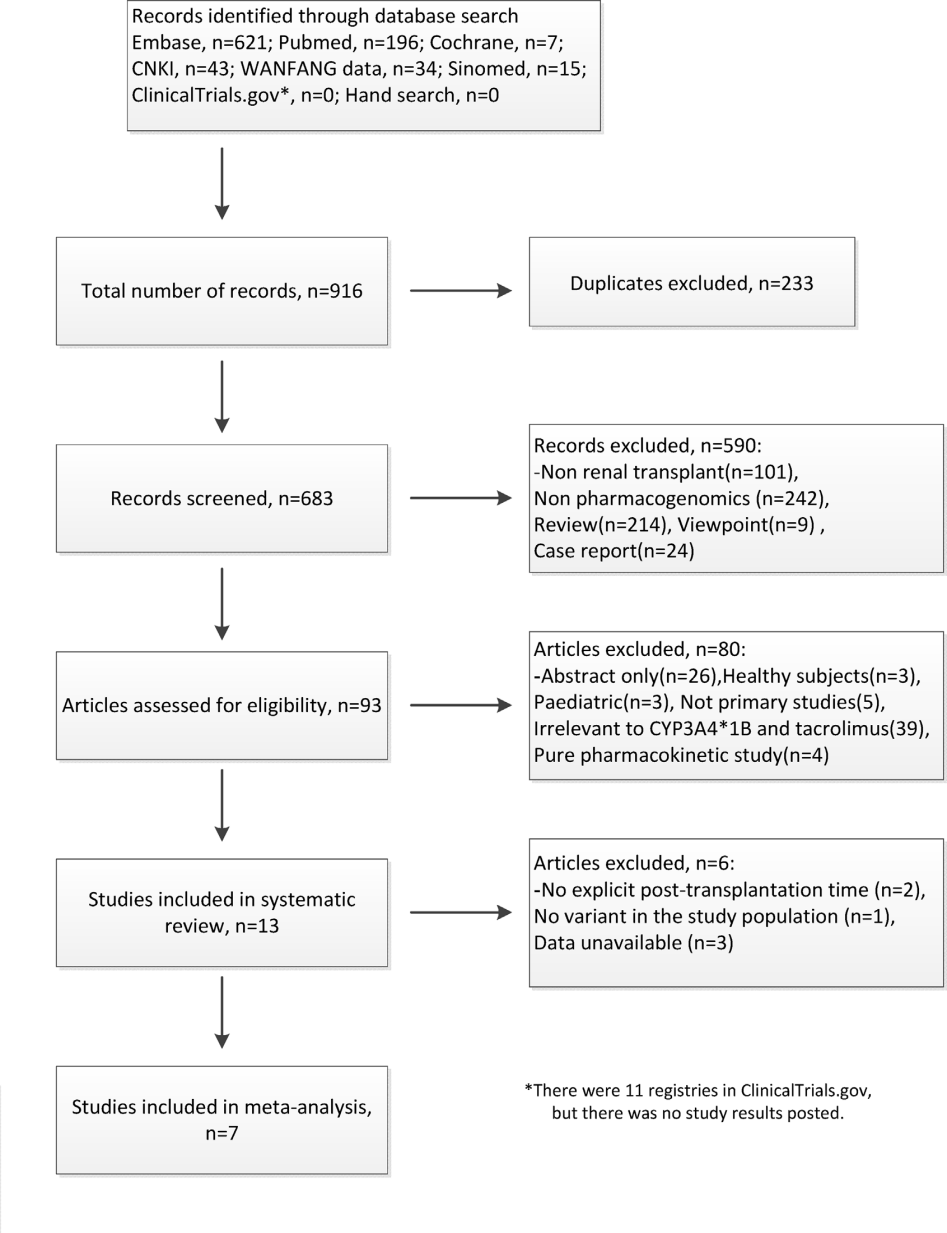
Flow diagram of the systematic review.

A total of 683 publications were identified by the literature search. After screening the titles and abstracts, the full texts of the remaining 93 studies were further assessed, and 7 studies[19–25] were included in the final meta-analysis. The details of identification of the eligible studies and the reasons for the exclusion of studies are presented in Fig 1. All 7 studies were published in English. A total of 1182 adult renal transplant recipients were included in the studies, and the characteristics of the 7 included studies are presented in Table 1. The results of quality assessment are presented in Table 2. Five original authors[20,21,23–25] were contacted for missing or specific demographic data, and three authors[20,21,25] replied.

**Table 1:** Characteristics of the studies included in the meta-analysis.

Study	Cases/Male(n)	Location/Ethnicity	Age(years)	Immunosuppressive protocol ^a^	Initial dose of tacrolimus(mg/kg/day)	Desired trough concentration(ng/ml)	Hardy-Weinberg equilibrium	Allele frequencies of *CYP3A4* (%)	
								*1	*1B
**Kurzawski 2014** ^**[**^ ^**19**^ ^**]**^	241/134	Poland/Caucasian	45.8+/-12.4	Tac+MMF+steroids	0.1	1^st^ month:10–15;Subsequent:8–10	Yes	96.9	3.1
**Tavira 2013** ^**[**^ ^**20**^ ^**]**^	206/126	Spain/Caucasian	48.6+/-13.5	Tac+MMF+prednisone	0.2	0–3 months:10–15;Subsequent:5–15	Yes	97.1	2.9
**Gervasini 2012** ^**[**^ ^**21**^ ^**]**^	103/62	Spain/Caucasian	48.7+/-16.9	Tac+MMF+steroids	0.2	0–3 months:10–15;Subsequent:5–10 ^b^	Yes	97.6	2.4
**Tavira 2011** ^**[**^ ^**22**^ ^**]**^	400/242	Spain/Caucasian	48.02+/-13.29	Tac+MMF+prednisone	0.2	0–3 months:10–15;Subsequent:5–10	Yes	96.9	3.1
**Singh 2009** ^**[**^ ^**23**^ ^**]**^	73/NA	India/North Indian	NA	Tac+MMF/Aza+prednisolone	0.16	1^st^ month: 10–12;3^rd^ month:8–10	No	97.3	2.7
**Kuypers 2007** ^**[**^ ^**24**^ ^**]**^	95/57	Belgium/Caucasian	51.3+/-14.1	Tac+MMF+methylprednisolone	0.2	8–15	Yes	96.3	3.7
**Hesselink 2003** ^**[**^ ^**25**^ ^**]**^	64/34	Netherlands/Asian,Black & White	NA	NA	0.2–0.3	NA	No	89.8	10.2

NA: not available.

^a^Tac: tacrolimus; MMF: mycophenolate mofetil; Aza: Azathioprine.

^b^Between October 2001 and February 2003, 7–15 ng/ml between June 2000 and September 2001.

**Table 2:** Quality assessment of the studies included in the meta-analysis.

First author	Year	Clear statement of background, objectives and hypothesis	Describe the studies information	Clear eligibility criteria	Clear definition of variables	Credible method of concentration measured	Credible genetic testing method	Replicability of statistical methods	Assessment of H-W equilibrium	Sufficient descriptive demographic data	Report the withdrew person and reasons	Statement of outcome data	Funding
**Kurzawski, M**	2014	+	+	+	+	+	+	+	+	+	±	+	+
**Tavira, B**	2013	+	±	+	+	+	+	+	+	±	+	+	+
**Gervasini, G**	2012	+	+	+	+	+	+	+	+	+	+	+	+
**Tavira, B**	2011	+	+	+	+	+	+	+	+	+	+	+	+
**Singh, R**	2009	+	+	+	+	+	+	+	-	±	+	+	+
**Kuypers, D R J**	2007	+	+	+	+	+	+	+	+	+	±	+	+
**Hesselink, D A**	2003	+	±	+	+	+	+	+	-	±	+	+	+

“+”: detailed description; “±”: incomplete description; “-”: no description.

### Effects of the CYP3A4*1B genetic polymorphism on the weight-adjusted tacrolimus dose (Dose)

All 7 studies[19–25] evaluated the association between the CYP3A4*1B genetic polymorphism and weight-adjusted tacrolimus daily dose (Dose) at different post-transplantation time. The result of the meta-analysis revealed that CYP3A4*1/*1 recipients required a lower Dose than CYP3A4*1B carriers (WMD -0.047; 95% CI -0.062 ~ -0.031; *P* < 0.001). However, substantial heterogeneity existed (*I* ^2^ = 75.3%), and a meta-regression was performed to explore the sources of heterogeneity with respect to the following factors: post-transplantation time, ethnicity, location, method of concentration measured, initial dose and Hardy-Weinberg equilibrium. The results of the meta-regression are presented in Table 3. The tacrolimus daily dose varied by post-transplantation time, ethnicity and location, although only “method of concentration measured” (*r* = 0.027, *P* = 0.008) and “initial dose” (*r* = -0.077, *P* < 0.001) contributed to heterogeneity. We performed subgroup analyses for all of the above covariates with the exception of the “Hardy-Weinberg equilibrium” because of collinearity, and the results are presented in Table 4. As Table 4 indicated, although the heterogeneity persisted, most subgroups demonstrated that CYP3A4*1/*1 recipients required a lower Dose than CYP3A4*1B carriers, but there was no statistical significance detected in subgroup analyses by post-transplantation time (1 month & 3 months), ethnicity (North Indian), location (India) or initial dose(0.1–0.16 mg/kg/day) involving the India population.

**Table 3:** Results of the meta-regression in all included renal transplant recipients. ^a^

Subjects	Covariates	*r*	*P*	Tau^2^	*I* ^*2*^-res	Adjusted R^2^
**Dose**	post-transplantation time	-0.005	0.177	0.000134	19.47%	86.02%
**Dose**	ethnicity	-0.011	0.280	0.000134	19.47%	86.02%
**Dose**	location	0.036	0.083	0.000134	19.47%	86.02%
**Dose**	method ^b^	0.027	0.008	0.000134	19.47%	86.02%
**Dose**	initial dose	-0.077	< 0.001	0.000134	19.47%	86.02%
**C**_**0**_ **/Dose**	post-transplantation time	13.320	< 0.001	0	0.00%	100.00%
**C**_**0**_ **/Dose**	ethnicity	-39.806	< 0.001	0	0.00%	100.00%
**C**_**0**_ **/Dose**	location	-48.820	0.099	0	0.00%	100.00%
**C**_**0**_ **/Dose**	method*	57.668	< 0.001	0	0.00%	100.00%
**C**_**0**_ **/Dose**	initial dose	-85.672	< 0.001	0	0.00%	100.00%

*r*: coefficient of correlation; Tau^2^: REML estimate of between-study variance; *I* ^2^-res: residual variation due to heterogeneity; Adjusted R^2^: proportion of between-study variance explained.

^a^ covariate “Hardy-Weinberg equilibrium” dropped because of collinearity.

^b^ method: method of concentration measured;

*method: method of concentration measured.

**Table 4:** Subgroup analysis of the CYP3A4*1B genetic polymorphism on weight adjusted tacrolimus daily dose (Dose) and C_0_/Dose by various factors in all included renal transplant recipients.

Subgroup(CYP3A4*1/*1 vs. CYP3A4*1B carriers)	Studies included (observations)	WMD(95% CI)	*P*	*I* ^*2*^(%)	SMD(95% CI)	*P*	*I* ^*2*^(%)
**Dose**							
**By time of PT** ^**c**^							
7 days	19–22,24(5)	-0.048(-0.083,-0.014)	**0.006**	78.2	-0.781(-1.281,-0.281)	**0.002**	69.5
1 month	19,21,23(3)	-0.012(-0.036,0.012)	0.322	34.8	-0.204(-0.626,0.217)	0.342	0.0
3 months	19,23-25(4)	-0.043(-0.092,0.007)	0.091	82.8	-0.928(-1.812,-0.044)	**0.040**	81.2
6 months	19–22,24(5)	-0.058(-0.081,-0.036)	**<0.001**	43.4	-1.033(-1.465,-0.601)	**<0.001**	58.6
12 months	19,21,22,24,25(5)	-0.061(-0.096,-0.027)	**0.001**	79.3	-1.241(-1.973,-0.508)	**0.001**	84.2
**By ethnicity**							
Caucasian	19–22,24(18)	-0.051(-0.067,-0.035)	**<0.001**	69.5	-0.932(-1.214,-0.650)	**<0.001**	73.4
North Indian	23(2)	0.010(-0.010,0.030)	0.330	0.0	0.252(-0.566,1.070)	0.546	0.0
Mixed^#^	25(2)	-0.066(-0.094,-0.038)	**<0.001**	0.0	-1.356(-1.863,-0.850)	**<0.001**	0.0
**By location**							
Europe	19–22,24,25(20)	-0.052(-0.067,-0.038)	**<0.001**	67.3	-0.971(-1.236,-0.707)	**<0.001**	72.1
India	23(2)	0.010(-0.010,0.030)	0.330	0.0	-0.252(-0.566,1.070)	0.546	0.0
By method*							
EMIT	21,25(6)	-0.046(-0.067,-0.025)	**<0.001**	51.7	-1.103(-1.530,-0.675)	**<0.001**	36.2
MEIA	19,23,24(11)	-0.031(-0.052,-0.009)	**0.005**	71.1	-0.837(-1.332,-0.342)	**0.001**	81.9
CMIA	20,22(5)	-0.074(-0.100,-0.049)	**<0.001**	65.2	-0.873(-1.155,-0.592)	**<0.001**	44.4
**By initial dose**							
0.2–0.3mg/kg/day	20–22,24,25(15)	-0.065(-0.080,-0.049)	**<0.001**	59.5	-1.203(-1.501,-0.906)	**<0.001**	66.7
0.1–0.16mg/kg/day	19,23(7)	-0.008(-0.020,0.004)	0.204	0.0	-0.322(-0.553,-0.090)	**0.006**	0.0
**C**_**0**_ **/Dose**							
**By time of PT** ^**c**^							
7 days	19-22(4)	19.971(-7.707,47.650)	0.157	81.1	0.189(-0.156,0.533)	0.283	26.6
1 month	19,21,23(3)	34.966(-6.988,76.920)	0.102	79.4	0.247(-0.738,1.232)	0.623	75.4
3 months	19,23,25(3)	7.676(-20.134,35.485)	0.589	0.0	0.151(-0.239,0.541)	0.448	0.0
6 months	19-22(4)	52.588(22.387,82.789)	**0.001**	59.1	0.344(-0.026,0.715)	0.069	41.6
12 months	19,21,22,25(4)	62.219(14.218,110.221)	**0.011**	86.5	0.350(0.037,0.663)	**0.028**	14.0
**By ethnicity**							
Caucasian	19-22(14)	47.245(26.341,68.150)	**<0.001**	85.1	0.335(0.160,0.510)	**<0.001**	23.7
North Indian	23(2)	-42.457(-91.044,6.131)	0.087	0.0	-0.821(-1.645,0.002)	0.051	0.0
Mixed ^a^	25(2)	15.651(-9.785,41.087)	0.228	0.0	0.179(-0.299,0.657)	0.462	0.0
**By method** ^**b**^							
EMIT	21,25(6)	62.270(28.801,95.739)	**<0.001**	86.1	0.692(0.276,1.107)	**0.001**	35.4
MEIA	19,23(7)	23.487(-2.353,49.327)	0.075	50.1	0.230(-0.060,0.519)	0.120	28.7
CMIA	20,22(5)	24.973(-0.538,50.484)	0.055	80.0	0.120(-0.084,0.323)	0.248	0.0
**By initial dose**							
0.2–0.3mg/kg/day	20–22,25(11)	45.395(20.373,70.417)	**<0.001**	88.9	0.326(0.094,0.559)	**0.006**	38.0
0.1–0.16mg/kg/day	19,23(7)	23.487(-2.353,49.327)	0.075	50.1	0.230(-0.060,0.519)	0.120	28.7

^a^ Mixed: Asian, Black & White.

^b^ method: method of concentration measured;

^c^ PT: post-transplantation.

Considering the above results, we removed the study involving the Indian population[23] and performed the subgroup analysis according to post-transplantation time. Unlike before, the 1 month (WMD -0.023; 95% CI -0.045 ~ -0.000; *P* = 0.047; *I* ^*2*^ = 0.0%) and 3 months (WMD -0.065; 95% CI -0.119 ~ -0.010; *P* = 0.021; *I* ^*2*^ = 70.4%) subgroups exhibited significant difference between CYP3A4*1/*1 and CYP3A4*1B carriers (Table 5). Thus, in all included populations, CYP3A4*1/*1 recipients required lower Dose than CYP3A4*1B carriers at 7 days, 6 months and 12 months post-transplantation; in European recipients (mostly Caucasian), CYP3A4*1/*1 recipients required lower Dose than CYP3A4*1B carriers within the first year post-transplantation.

**Table 5:** Subgroup analysis of the CYP3A4*1B genetic polymorphism on weight adjusted tacrolimus daily dose (Dose) and C_0_/Dose by time of post-transplantation in European (Indian population removed).

Subgroup(CYP3A4*1/*1 vs. CYP3A4*1B carriers)	Studies included (observations)	WMD(95% CI)	*P*	*I* ^*2*^(%)	SMD(95% CI)	*P*	*I* ^*2*^(%)
**Dose**							
**By time of PT** ^**c**^							
7 days	19–22,24(5)	-0.048(-0.083,-0.014)	**0.006**	78.2	-0.781(-1.281,-0.281)	**0.002**	69.5
1 month	19,21(2)	-0.023(-0.045,-0.000)	**0.047**	0.0	-0.274(-0.727,0.179)	0.235	0.0
3 months	19,24,25(3)	-0.065(-0.119,0.010)	**0.021**	70.4	-1.231(-2.188,-0.273)	**0.012**	83.0
6 months	19–22,24(5)	-0.058(-0.081,-0.036)	**<0.001**	43.4	-1.033(-1.465,-0.601)	**<0.001**	58.6
12 months	19,21,22,24,25(5)	-0.061(-0.096,-0.027)	**0.001**	79.3	-1.241(-1.973,-0.508)	**0.001**	84.2
**By *CYP3A5* & time of PT** ^**c**^							
CYP3A5*3/*3							
7 days	20,22(2)	-0.031(-0.059,-0.003)	**0.033**	13.2	-0.444(-0.818,-0.071)	**0.020**	0.0
6 months	20,22(2)	-0.037(-0.050,-0.024)	**<0.001**	0.0	-0.604(-1.005,-0.204)	**0.003**	10.6
12 months	22(1)^a^	-0.040(-0.064,-0.016)	**0.001**	NA	-0.278(-0.908,0.352)	0.388	NA
CYP3A5*1 carriers							
7 days	20–22,24(4)	-0.059(-0.141,0.022)	0.154	88.7	-0.793(-1.800,0.215)	0.123	76.9
1 month	21(1)^a^	0.020(-0.024,0.064)	0.371	NA	0.566(-0.705,1.836)	0.383	NA
3 months	24(1)^a^	-0.030(-0.145,0.085)	0.608	NA	-0.272(-1.292,0.748)	0.601	NA
6 months	20–22,24(4)	-0.032(-0.059,-0.004)	**0.025**	1.7	-0.622(-1.054,-0.190)	**0.005**	0.0
12 months	21,22,24(3)	-0.050(-0.092,-0.008)	**0.020**	47.7	-0.991(-1.557,-0.424)	**0.001**	17.2
**C**_**0**_ **/Dose**							
**By time of PT** ^**c**^							
7 days	19-22(4)	19.971(-7.707,47.650)	0.157	81.1	0.189(-0.156,0.533)	0.283	26.6
1 month	19,21(2)	58.129(40.584,75.675)	**<0.001**	0.0	0.643(0.176,1.109)	**0.007**	3.0
3 months	19,25(2)	14.790(-15.089,44.668)	0.332	0.0	0.244(-0.170,0.658)	0.247	0.0
6 months	19-22(4)	52.588(22.387,82.789)	**0.001**	59.1	0.344(-0.026,0.715)	0.069	41.6
12 months	19,21,22,25(4)	62.219(14.218,110.221)	**0.011**	86.5	0.350(0.037,0.663)	**0.028**	14.0
**By *CYP3A5* & time of PT** ^**c**^							
CYP3A5*3/*3							
7 days	19,20,22(3)	-5.800(-17.709,6.109)	0.340	0.0	-0.075(-0.490,0.340)	0.723	0.0
1 month	19(1)^a^	25.100(-8.015,58.215)	0.137	NA	0.225(-0.586,1.036)	0.587	NA
3 months	19(1)^a^	-37.400(-178.178,103.378)	0.603	NA	-0.376(-1.188,0.435)	0.364	NA
6 months	19,20,22(3)	49.715(6.896,92.533)	**0.023**	54.0	0.192(-0.224,0.607)	0.366	0.0
12 months	19,22(2)	21.241(-72.999,115.480)	0.659	72.0	0.028(-0.495,0.551)	0.918	8.5
CYP3A5*1 carriers ^**b**^							
7 days	19–22,24(5)	NA	NA	NA	0.365(-0.021,0.750)	0.064	0.0
1 month	19,21(2)	NA	NA	NA	0.222(-0.523,0.966)	0.559	0.0
3 months	19,24(2)	NA	NA	NA	0.323(-0.369,1.015)	0.360	0.0
6 months	19–22,24(5)	NA	NA	NA	0.142(-0.390,0.674)	0.601	36.3
12 months	19,21,22,24(4)	NA	NA	NA	0.619(-0.031,1.269)	0.062	45.7

NA: not available.

^a^ only one observation.

^b^ the units of C_0_/Dose are different, so the data were pooled by SMD;

^c^ PT: post-transplantation.

### Effects of the CYP3A4*1B genetic polymorphism on the tacrolimus trough blood concentration/Dose ratio (C_0_/Dose ratio)

Six studies[19–23,25] evaluated the association between the CYP3A4*1B genetic polymorphism and the C_0_/Dose ratio at different post-transplantation times. The result of the meta-analysis revealed that CYP3A4*1/*1 recipients exhibited higher C_0_/Dose ratios than CYP3A4*1B carriers (WMD 37.127; 95% CI 18.202 ~ 56.051; *P* < 0.001). Similar to the above analysis, substantial heterogeneity existed (*I* ^2^ = 83.4%), and a meta-regression was performed to explore the sources of heterogeneity. As shown in Table 3, post-transplantation time (*r* = 13.320, *P* < 0.001), ethnicity (*r* = -39.806, *P* < 0.001), method of concentration measured (*r* = 57.668, *P* < 0.001) and initial dose (*r* = -85.672, *P* < 0.001) contributed to heterogeneity. Subgroup analyses of post-transplantation time, ethnicity, method of concentration measured and initial dose were performed. CYP3A4*1/*1 recipients exhibited higher C_0_/Dose ratios than CYP3A4*1B carriers in the subgroups of post-transplantation time (6 months & 12 months), ethnicity (Caucasian), method of concentration measured (EMIT) and initial dose (0.2–0.3 mg/kg/day) (Table 4), but the heterogeneity persisted in some subgroups.

When the study concerning the India population was removed, the results of the 1 month post-transplantation exhibited significant differences (WMD 58.129; 95% CI 40.584 ~ 75.675; *P* < 0.001; *I* ^*2*^ = 0.0%) (Table 5). Thus, in all included population, CYP3A4*1/*1 recipients exhibited higher C_0_/Dose ratios than CYP3A4*1B carriers at 6 and 12 months; in European recipients (mostly Caucasian), CYP3A4*1/*1 recipients exhibited higher C_0_/Dose ratios than CYP3A4*1B carriers at 1 month, 6 months and 12 months post-transplantation.

### Effects of the CYP3A4*1B genetic polymorphisms on Dose and the C_0_/Dose ratio stratified by the *CYP3A5* genotype

We performed subgroup analyses at different post-transplantation times stratified by the *CYP3A5* genotype. In CYP3A5*3/*3 recipients, CYP3A4*1/*1 recipients required lower Dose than CYP3A4*1B carriers at 7 days, 6 months and 12 months post-transplantation; in CYP3A5*1 carriers, CYP3A4*1/*1 recipients required lower Dose than CYP3A4*1B carriers at 6 months and 12 months post-transplantation. Except for CYP3A5*3/*3 recipients at the time point of 6 months post-transplantation, there was no significant difference in the C_0_/Dose ratio between the CYP3A4*1/*1 recipients and CYP3A4*1B carriers between different *CYP3A5* genotypes (Table 5). The results suggested that the effect of the CYP3A4*1B genetic polymorphism on tacrolimus pharmacokinetics was independent at 7 days, 6 months and 12 months post-transplantation in CYP3A5*3/*3 recipients; and at 6 months and 12 months post-transplantation in CYP3A5*1 carriers.

## Discussion

The findings of our meta-analysis suggest that the CYP3A4*1B genetic polymorphism influences the weight-adjusted tacrolimus daily dose and the C_0_/Dose ratio in adult renal transplant recipients.

In all included renal transplant recipients, relative to CYP3A4*1B carriers, CYP3A4*1/*1 recipients required a lower weight-adjusted tacrolimus daily dose (Dose) at 7 days, 6 months and 12 months post-transplantation, and exhibited a higher C_0_/Dose ratio at 6 and 12 months post-transplantation. This finding suggests that CYP3A4*1/*1 recipients required a lower initial dose within 7 days post-transplantation and a lower maintenance dose to achieve the target blood concentration relative to CYP3A4*1B carriers at 6 and 12 months post-transplantation.

In the meta-regression and subgroup analyses, we determined that the ethnicity and location influenced the pooled estimate results significantly. Therefore, the meta-analysis was stratified by post-transplantation time and was performed in the European recipients. The results revealed that CYP3A4*1/*1 recipients exhibited a lower weight-adjusted tacrolimus daily dose (Dose) within the entire first year and a higher C_0_/Dose ratio at 1 month, 6 months and 12 months post-transplantation compared with CYP3A4*1B carriers. Thus, the CYP3A4*1B genetic polymorphism plays a more important role in European renal transplant recipients. The results of the sensitivity analyses were consistent with the meta-regression results; excluding the Indian population changed the pooled estimate at 1 month and 3 months with respect to Dose and at 1 month with respect to the C_0_/Dose ratio post-transplantation, but there was no effect on the overall estimate. Although there was no significant difference in the C_0_/Dose ratio between CYP3A4*1/*1 recipients and CYP3A4*1B carriers stratified by *CYP3A5* genotype, the dose requirement differed significantly at 6 and 12 months post-transplantation, which indicated that different doses were required in CYP3A4*1/*1 recipients and CYP3A4*1B carriers stratified by the *CYP3A5* genotype; these data further indicate that CYP3A4*1B and CYP3A5*3 may have independent effects on tacrolimus pharmacokinetics.

In clinical settings, the initial tacrolimus dose is given according to the weight of different renal recipients, and the maintenance dose is adjusted according to the blood concentration[26]. Acute rejection and nephrotoxicity are unavoidable, especially in the early post-transplantation stage because the clinicians must adjust the dose frequently to achieve the target blood concentration. According to our meta-analysis, the CYP3A4*1B genetic polymorphism should be considered when determining the initial tacrolimus dose and adjusting the maintenance dose, which may be helpful to achieve the target concentration in a shorter time and reduce the concentration fluctuations. Because of the therapeutic drug monitoring (TDM), even though the CYP3A4*1B carriers required a higher Dose than the CYP3A4*1/*1 recipients, the C_0_/Dose ratio were not stable within 3 months post-transplantation.

Several limitations should be noted in our meta-analysis. First, only 7 observational studies (involving 1182 adult renal transplant recipients) were included, and there were only 77 CYP3A4*1B carriers. Considering the influence of large sample size from one single study, a sensitivity analysis had been performed to assess the validity of the pooled estimates. There was no single study which have a significant influence on the pooled estimate except for the study in the Indian population, which had been analyzed in Results. And our meta-analysis had tried to include all the studies that evaluated the effects of the CYP3A4*1B genetic polymorphism on tacrolimus pharmacokinetics, which could be retrieved from electronic database at present. The limitation of sample size influenced the pooled estimate possibly. Because of only 7 studies were included, the publication bias analysis was not performed. Second, the formulae for estimating the mean using the values of the median provided by Hozo *et al*. introduced some uncertainty [14], and the formulae for combining groups provided by the Cochrane handbook 5.1.0 Table 7.7.a provided a slight underestimate of the desired standard deviation [18]; bias resulting from the methods is unavoidable. Third, the results of our meta-analysis should be interpreted with caution because of the substantial heterogeneity across all current available studies, even though we performed meta-regression to explore the source of heterogeneity and subgroup analyses to minimize the heterogeneity. According to our meta-regression results, the method of concentration measured was the source of heterogeneity, because different methods of concentration measured have different properties in the cross-reaction with tacrolimus metabolites[27], a further subgroup analyses by method of concentration measured revealed that CYP3A4*1B carriers required higher Dose in all three subgroups, and had lower C_0_^/^Dose in EMIT subgroup (Table 4). Although the included studies had stated the demographic information and immunosuppressive protocol, different steroid tapering schedules may have influenced on the pooled estimate due to the interaction between steroids and tacrolimus[28]. Therefore, we listed the SMD of the pooled estimate to minimize the influence of the method of concentration measured and the steroids tapering schedules in various studies, which should be considered when the conclusions were interpreted by different transplantation centers based on different situations.

Futhermore, even though several studies[13] had demonstrated linkage disequilibrium between CYP3A4*1B and CYP3A5*1, in the combined CYP3A4*1B/CYP3A5*1 genotype analysis, Gervasini, G *et al*.[21] and Chitnis, S D *et al*.[29] reported that CYP3A4*1/*1 recipients exhibited higher tacrolimus trough concentrations and C_0_/Dose ratios than CYP3A4*1B carriers in *CYP3A5* expressers (CYP3A5*1/*1 or CYP3A5*1/*3); Tavira, B *et al*.[22] reported that, relative to CYP3A4*1B carriers stratified by *CYP3A5* (expressers or non-expressers respectively), CYP3A4*1/*1 recipients required lower tacrolimus doses, and exhibited higher tacrolimus trough concentrations and C_0_/dose ratios, which suggests a significant role of the CYP3A4*1B. Our meta-analysis revealed the CYP3A4*1B and CYP3A5*3 may have independent effects on tacrolimus pharmacokinetics. Furthermore, tacrolimus is a substrate for both P-glycoprotein (P-gp, coded by *ABCB1*) and CYP3A, *ABCB1* genetic polymorphisms (such as *C3435T*) have an influence on the activity of P-gp and tacrolimus pharmacokinetics[30]. The intestinal CYP3A4 and P-gp work together in a coordinated manner to serve as an absorption barrier against tacrolimus [31], and the altered activity of P-gp has a significant influence on tacrolimus metabolism by CYP3A4 in both gut and liver[32,33]. However, lack of studies focused on the interaction between *ABCB1* and *CYP3A4 g*enetic polymorphisms on tacrolimus in reanl transplant recipients, which limited analyzing the issues further. It is possible that a combination of *CYP3A4/5*[34] and *ABCB1* genotypes would be more helpful in making predictions than any single gene.

In conclusion, our meta-analysis suggests that the CYP3A4*1B genetic polymorphism may affect the tacrolimus dose requirements and the C_0_/Dose ratio within the first year post-transplantation in adult renal transplant recipients, especially in European recipients (mostly Caucasian). *CYP3A4* genotyping before transplantation would be helpful to provide an appropriate initial dose and adjust the maintenance dose in adult renal transplant recipients.

## Supporting Information

S1 Checklist PRISMA checklist. (DOC)

S2 Checklist Meta-analysis on genetic association studies form checklist. (DOCX)

S1 File Excluded articles list. (DOCX)

S2 File PRISMA flow diagram. (DOC)

## References

[1] Meier-Kriesche HU, Li S, Gruessner RW, Fung JJ, Bustami RT, Barr ML, et al (2006) Immunosuppression: evolution in practice and trends, 1994–2004. Am J Transplant 6: 1111–1131. 16613591 10.1111/j.1600-6143.2006.01270.x

[2] Kershner RP, Fitzsimmons WE (1996) Relationship of FK506 whole blood concentrations and efficacy and toxicity after liver and kidney transplantation. Transplantation 62: 920–926. 8878385 10.1097/00007890-199610150-00009

[3] Robles-Piedras AL, Gonzalez-Lopez EH (2009) Tacrolimus levels in adult patients with renal transplant. Proc West Pharmacol Soc 52: 33–34. 22128417

[4] Kahan BD, Keown P, Levy GA, Johnston A (2002) Therapeutic drug monitoring of immunosuppressant drugs in clinical practice. Clinical Therapeutics 24: 330–350. 11952020 10.1016/s0149-2918(02)85038-x

[5] Staatz CE, Tett SE (2004) Clinical Pharmacokinetics and Pharmacodynamics of Tacrolimus in Solid Organ Transplantation. Clinical Pharmacokinetics 43: 623–653. 15244495 10.2165/00003088-200443100-00001

[6] Hronova K, Sima M, Svetlik S, Matouskova O, Slanar O (2014) Pharmacogenetics and immunosuppressive drugs. Expert Rev Clin Pharmacol 7: 821–835. 10.1586/17512433.2014.966811 25301406

[7] Shiraga T, Matsuda H, Nagase K, Iwasaki K, Noda K, Yamazaki H, et al (1994) Metabolism of FK506, a potent immunosuppressive agent, by cytochrome P450 3A enzymes in rat, dog and human liver microsomes. Biochem Pharmacol 47: 727–735. 7510480 10.1016/0006-2952(94)90136-8

[8] Lampen A, Christians U, Guengerich FP, Watkins PB, Kolars JC, Bader A, et al (1995) Metabolism of the immunosuppressant tacrolimus in the small intestine: cytochrome P450, drug interactions, and interindividual variability. Drug Metab Dispos 23: 1315–1324. 8689938

[9] Tuteja S, Alloway RR, Johnson JA, Gaber AO (2001) The effect of gut metabolism on tacrolimus bioavailability in renal transplant recipients. Transplantation 71: 1303–1307. 11397967 10.1097/00007890-200105150-00021

[10] Tang HL, Xie HG, Yao Y, Hu YF (2011) Lower tacrolimus daily dose requirements and acute rejection rates in the CYP3A5 nonexpressers than expressers. Pharmacogenet Genomics 21: 713–720. 10.1097/FPC.0b013e32834a48ca 21886016

[11] Rojas L, Neumann I, Herrero MJ, Boso V, Reig J, Poveda JL, et al (2014) Effect of CYP3A5*3 on kidney transplant recipients treated with tacrolimus: a systematic review and meta-analysis of observational studies. Pharmacogenomics J. 10.1038/tpj.2014.38 25201288

[12] Terrazzino S, Quaglia M, Stratta P, Canonico PL, Genazzani AA (2012) The effect of CYP3A5 6986A>G and ABCB1 3435C>T on tacrolimus dose-adjusted trough levels and acute rejection rates in renal transplant patients: a systematic review and meta-analysis. Pharmacogenet Genomics 22: 642–645. 10.1097/FPC.0b013e3283557c74 22786571

[13] Hesselink DA, Bouamar R, Elens L, van Schaik RH, van Gelder T (2014) The role of pharmacogenetics in the disposition of and response to tacrolimus in solid organ transplantation. Clin Pharmacokinet 53: 123–139. 10.1007/s40262-013-0120-3 24249597

[14] Hozo SP, Djulbegovic B, Hozo I (2005) Estimating the mean and variance from the median, range, and the size of a sample. BMC Med Res Methodol 5: 13 15840177 10.1186/1471-2288-5-13 PMC1097734

[15] Little J, Higgins JP, Ioannidis JP, Moher D, Gagnon F, von Elm E, et al (2009) STrengthening the REporting of Genetic Association Studies (STREGA): an extension of the STROBE statement. PLoS Med 6: e22 10.1371/journal.pmed.1000022 19192942 PMC2634792

[16] Zhou X, Qian W, Li J, Zhang P, Yang Z, Wu L. (2013) Who are at risk for thromboembolism after arthroplasty? A systematic review and meta-analysis. Thrombosis research 132: 531–536. 10.1016/j.thromres.2013.09.005 24074702

[17] Terrazzino S, Cargnin S, Del RM, Danesi R, Canonico PL, Genazzani AA. (2013) DPYD IVS14+1G>A and 2846A>T genotyping for the prediction of severe fluoropyrimidine-related toxicity: a meta-analysis. Pharmacogenomics 14: 1255–1272. 10.2217/pgs.13.116 23930673

[18] Higgins JPT GSE (2011) Cochrane Handbook for Systematic Reviews of Interventions Version 5.1.0 [updated March 2011]. The Cochrane Collaboration.

[19] Kurzawski M, Dabrowska J, Dziewanowski K, Domanski L, Peruzynska M, Drozdzik M. (2014) CYP3A5 and CYP3A4, but not ABCB1 polymorphisms affect tacrolimus dose-adjusted trough concentrations in kidney transplant recipients. Pharmacogenomics 15: 179–188. 10.2217/pgs.13.199 24444408

[20] Tavira B, Coto E, Diaz-Corte C, Alvarez V, Lopez-Larrea C, Ortega F. (2013) A search for new CYP3A4 variants as determinants of tacrolimus dose requirements in renal-transplanted patients. Pharmacogenetics and Genomics 23: 445–448. 10.1097/FPC.0b013e3283636856 23778326

[21] Gervasini G, Garcia M, MacIas RM, Cubero JJ, Caravaca F, Benitez J. (2012) Impact of genetic polymorphisms on tacrolimus pharmacokinetics and the clinical outcome of renal transplantation. Transplant International 25: 471–480. 10.1111/j.1432-2277.2012.01446.x 22369694

[22] Tavira B, Garcia EC, Diaz-Corte C, Ortega F, Arias M, Torres A, et al (2011) Pharmacogenetics of tacrolimus after renal transplantation: Analysis of polymorphisms in genes encoding 16 drug metabolizing enzymes. Clinical Chemistry and Laboratory Medicine 49: 825–833. 10.1515/CCLM.2011.143 21480817

[23] Singh R, Srivastava A, Kapoor R, K. Sharma R, D. Mittal R (2009) Impact of CYP3A5 and CYP3A4 gene polymorphisms on dose requirement of calcineurin inhibitors, cyclosporine and tacrolimus, in renal allograft recipients of North India. Naunyn-Schmiedeberg's Archives of Pharmacology 380: 169–177. 10.1007/s00210-009-0415-y 19343327

[24] Kuypers DRJ, De Jonge H, Naesens M, Lerut E, Verbeke K, Vanrenterghem Y. (2007) CYP3A5 and CYP3A4 but not MDR1 single-nucleotide polymorphisms determine long-term tacrolimus disposition and drug-related nephrotoxicity in renal recipients. Clinical Pharmacology and Therapeutics 82: 711–725. 17495880 10.1038/sj.clpt.6100216

[25] Hesselink DA, Van Schaik RHN, Van Der Heiden IP, Van Der Werf M, Smak Gregoor PJH, Lindemans J, et al (2003) Genetic polymorphisms of the CYP3A4, CYP3A5, and MDR-1 genes and pharmacokinetics of the calcineurin inhibitors cyclosporine and tacrolimus. Clinical Pharmacology and Therapeutics 74: 245–254. 12966368 10.1016/S0009-9236(03)00168-1

[26] Kidney Disease: Improving Global Outcomes (KDIGO) Transplant Work Group (2009) KDIGO clinical practice guideline for the care of kidney transplant recipients. Am J Transplant 9 Suppl 3: S1–S155. 10.1111/j.1600-6143.2009.02834.x 19845597

[27] Hashi S, Masuda S, Kikuchi M, Uesugi M, Yano I, Omura T, et al (2014) Assessment of four methodologies (microparticle enzyme immunoassay, chemiluminescent enzyme immunoassay, affinity column-mediated immunoassay, and flow injection assay-tandem mass spectrometry) for measuring tacrolimus blood concentration in Japanese liver transplant recipients. Transplant Proc 46: 758–760. 10.1016/j.transproceed.2013.11.060 24767342

[28] Ferraris JR, Argibay PF, Costa L, Jimenez G, Coccia PA, Ghezzi LF, et al (2011) Influence of CYP3A5 polymorphism on tacrolimus maintenance doses and serum levels after renal transplantation: age dependency and pharmacological interaction with steroids. Pediatr Transplant 15:525–532. 10.1111/j.1399-3046.2011.01513.x 21711429

[29] Chitnis SD, Ogasawara K, Schniedewind B, Gohh RY, Christians U, Akhlaghi F. (2013) Concentration of tacrolimus and major metabolites in kidney transplant recipients as a function of diabetes mellitus and cytochrome P450 3A gene polymorphism. Xenobiotica 43: 641–649. 10.3109/00498254.2012.752118 23278282 PMC4116556

[30] Liu YY, Li C, Cui Z, Fu X, Zhang S, Fan LL, et al (2013) The effect of ABCB1 C3435T polymorphism on pharmacokinetics of tacrolimus in liver transplantation: a meta-analysis. Gene 531: 476–488. 10.1016/j.gene.2013.09.024 24042126

[31] Zhang Y, Benet LZ (2001) The gut as a barrier to drug absorption: combined role of cytochrome P450 3A and P-glycoprotein. Clin Pharmacokinet 40: 159–168. 11327196 10.2165/00003088-200140030-00002

[32] Benet LZ, Cummins CL, Wu CY (2004) Unmasking the dynamic interplay between efflux transporters and metabolic enzymes. Int J Pharm 277: 3–9. 15158963 10.1016/j.ijpharm.2002.12.002

[33] Wu CY, Benet LZ (2003) Disposition of tacrolimus in isolated perfused rat liver: influence of troleandomycin, cyclosporine, and gg918. Drug Metab Dispos 31: 1292–1295. 14570757 10.1124/dmd.31.11.1292

[34] Rojas L, Neumann I, Herrero MJ, Boso V, Reig J, Poveda JL, et al (2014) Effect of CYP3A5*3 on kidney transplant recipients treated with tacrolimus: a systematic review and meta-analysis of observational studies. Pharmacogenomics J 15: 38–48 10.1038/tpj.2014.38 25201288

