# Supplementary material for: Effects of the CYP3A4*1B Genetic Polymorphism on the Pharmacokinetics of Tacrolimus in Adult Renal Transplant Recipients: A Meta-Analysis
Source: PLoS One. 2015 Jun 3;10(6):e0127995. doi: 10.1371/journal.pone.0127995 (PMC4454552; doi:10.1371/journal.pone.0127995)
Supplement: S2 Checklist — (DOCX) [file pone.0127995.s002.docx]

**Meta-analysis on Genetic Association Studies Checklist | PLOS ONE**

|  | Item | Section name and paragraph number within manuscript |
| --- | --- | --- |
|  | **Introduction** |  |
| 1 | Provide a detailed justification for the polymorphism studied; if a single polymorphism was analyzed, give details as to why others were not included in the meta-analysis. | Introduction- Para 1 |
| 2 | Provide a detailed justification for the population(s) and clinical condition studied. | Introduction- Para 2 |
|  | **Methods** |  |
| 3 | Provide full details of the search strategy employed; outline the full electronic search strategy –specific combination of keywords and any limits applied- for at least one database. Specify whether synonyms of polymorphisms/genes (e.g. SNP number) were searched. | Methods-search strategy and study selection-Para 1 |
| 4 | Report full details on the inclusion and exclusion criteria applied for selecting studies.  *Please list the excluded articles and the reasons for exclusion of each article in a supplementary file.* | Methods-search strategy and study selection-Para 2 |
| 5 | Provide details on how the quality of the studies included in the analyses was assessed. | Methods-data extraction and quality assessment- Para 2 |
| 6 | Describe steps taken to contact study authors to identify additional studies and to request missing data. | Methods-search strategy and study selection- Para 1 |
| 7 | Describe how environmental effects were adjusted for, if this adjustment was not conducted, outline the reasons for this. | None. Because of the specificity of renal transplant recipients, the environment is complex. The included studies had excluded the recipients who would possibly be influenced by the environment, such as drug inter-actions. Besides, we performed meta-regression and subgroup analyses by ethnicity and location. |
| 8 | Describe the methods of handling heterogeneity/between-study variance. | Methods-statistical analysis- Para 2 |
| 9 | Describe how the Hardy-Weinberg equilibrium and linkage disequilibrium were assessed. | None. Because in the included studies, few mutant homozygote was found, so the mutant heterozygote and homozygote were trrated as mutant carriers. And not all included studies research the linkage disequilibrium, so we discuss it in "Discussion" |
| 10 | Describe and justify the choice of model for the analyses (per-allele vs per-genotype vs genetic model-free, random effects vs fixed effects). | Methods-statistical analysis- Para 1 |
| 11 | Describe whether a sensitivity analysis has been completed. | Methods-statistical analysis- Para 2 |
| 12 | Describe whether an assessment of the effects of population stratification has been conducted. | Methods-statistical analysis-Para 2 |
| 13 | Describe whether study-specific results have been assessed and if so the reasons for this (e.g. forest plot). | Figure 2&3 |
|  | **Results** |  |
| 14 | Include flow diagram for the studies included in the meta-analysis as the first figure for the manuscript | Results-Characteristics of the Articles Included in the Meta-analysis-Para 1 |
| 15 | Report details on allele/genotype prevalence. | Table 1 |
| 16 | Report the effect size estimates and p values for each analysis. | Table 4 and Results-Para 2-3 |
|  | **Discussion** |  |
| 17 | Discuss the limitations of the meta-analysis, including genotyping errors/bias and publication bias. | Discussion-Para 5 |
| 18 | If the meta-analysis identifies an association within a subgroup of the population studied but not another, discuss the implications of these results, and if applicable the possibility of subgroup-specific publication bias. | Discussion Para 4 |
| 19 | Discuss the suitability of the sample size employed to the research question and the power of the study. | Discussion-Para 5 |
