# Supplementary material for: Effects of the CYP3A4*1B Genetic Polymorphism on the Pharmacokinetics of Tacrolimus in Adult Renal Transplant Recipients: A Meta-Analysis
Source: PLoS One. 2015 Jun 3;10(6):e0127995. doi: 10.1371/journal.pone.0127995 (PMC4454552; doi:10.1371/journal.pone.0127995)
Supplement: S1 File — (DOCX) [file pone.0127995.s003.docx]

**Included(7)**

Kurzawski M, Dabrowska J, Dziewanowski K, Domanski L, Peruzynska M, et al. (2014) CYP3A5 and CYP3A4, but not ABCB1 polymorphisms affect tacrolimus dose-adjusted trough concentrations in kidney transplant recipients. Pharmacogenomics 15: 179-188.

Tavira B, Coto E, Diaz-Corte C, Alvarez V, Lopez-Larrea C, et al. (2013) A search for new CYP3A4 variants as determinants of tacrolimus dose requirements in renal-transplanted patients. Pharmacogenetics and Genomics 23: 445-448.

Gervasini G, Garcia M, MacIas RM, Cubero JJ, Caravaca F, et al. (2012) Impact of genetic polymorphisms on tacrolimus pharmacokinetics and the clinical outcome of renal transplantation. Transplant International 25: 471-480.

Tavira B, Garcia EC, Diaz-Corte C, Ortega F, Arias M, et al. (2011) Pharmacogenetics of tacrolimus after renal transplantation: Analysis of polymorphisms in genes encoding 16 drug metabolizing enzymes. Clinical Chemistry and Laboratory Medicine 49: 825-833.

Singh R, Srivastava A, Kapoor R, K. Sharma R, D. Mittal R (2009) Impact of CYP3A5 and CYP3A4 gene polymorphisms on dose requirement of calcineurin inhibitors, cyclosporine and tacrolimus, in renal allograft recipients of North India. Naunyn-Schmiedeberg's Archives of Pharmacology 380: 169-177.

Kuypers DRJ, De Jonge H, Naesens M, Lerut E, Verbeke K, et al. (2007) CYP3A5 and CYP3A4 but not MDR1 single-nucleotide polymorphisms determine long-term tacrolimus disposition and drug-related nephrotoxicity in renal recipients. Clinical Pharmacology and Therapeutics 82: 711-725.

Hesselink DA, Van Schaik RHN, Van Der Heiden IP, Van Der Werf M, Smak Gregoor PJH, et al. (2003) Genetic polymorphisms of the CYP3A4, CYP3A5, and MDR-1 genes and pharmacokinetics of the calcineurin inhibitors cyclosporine and tacrolimus. Clinical Pharmacology and Therapeutics 74: 245-254.

**NOTE: There are some articles in Chinese. And we list the excluded full-text articles and reasons (“Articles assessed for eligibility, n=93” in flow diagram), but do not list the excluded literatures by screening the titles and abstracts.**

**Abstract only (26)**

Guo YY, Jiang WT, Shen ZY, Zhang Y (2013) Study on the regularity between CYP3A5/3A4 polymorphism in medication of tacrolimus after renal transplantation. Acta Pharmacologica Sinica 34: 37.

Van Schaik RHN, Elens LE, Hesselink DA, Haufroid V, Van Gelder T (2013) Pharmacogenetics and immunosuppressive therapy in solid organ transplantation. Drug Metabolism and Drug Interactions 28: A4.

Ogasawara K, Chitnis SD, Gohh RY, Christians U, Akhlaghi F (2013) Multidrug resistance-associated protein 2 (MRP2) affects tacrolimus disposition in a haplotype-specific manner. Clinical Pharmacology and Therapeutics 93: S82.

李丹滢, 方芸 (2013) CYP3A4/5、ABCB1单倍型与中国肾移植患者他克莫司血药谷浓度相关性的研究. 2013年中国临床药学学术年会暨第九届临床药师论坛. 中国江西南昌. pp. 8.

CYP3A4活性、CYP3A5基因型和红细胞比容影响他克莫司剂量需求和体内清除. 今日药学 22: I3.

De Jonge H, De Loor H, Verbeke K, Vanrenterghem Y, Kuypers DR (2012) In vivo CYP3A4-activity, CYP3A5-genotype and hematocrit predict tacrolimus dose-requirements and clearance in renal transplant recipients. Transplantation 94: 248-249.

De Jonge H, De Loor H, Verbeke K, Vanrenterghem Y, Kuypers DR (2012) In vivo CYP3A4-activity and CYP3A5-genotype predict tacrolimus pharmacokinetics in renal transplant recipients. Clinical Pharmacology and Therapeutics 91: S49-S50.

De Jonge H, De Loor H, Verbeke K, Vanrenterghem Y, Kuypers DR (2012) Progressive decline in in vivo CYP3A4-activity explains time-related increase in dose corrected tacrolimus exposure after renal transplantation. Clinical Pharmacology and Therapeutics 91: S18.

郭媛媛, 张弋 (2012) CYP3A5/3A4多态性与肾移植患者术后他克莫司用药的变化规律. 2012中国器官移植大会. 中国福建厦门. pp. 1.

李丹滢, 陈燕华, 方芸 (2012) CYP3A4/5单倍型将影响他克莫司血药谷浓度. 第二届全国治疗药物监测学术年会. 中国北京. pp. 1.

徐东升, 王洪伟, 刘双德, 田川, 焉杰克, et al. (2012) CYP3A及MDR1基因表达与他克莫司血药浓度的相关性研究. 2012中国器官移植大会. 中国福建厦门. pp. 1.

Birdwell KA, Grady B, Choi L, Xu H, Denny JC, et al. (2011) Use of a novel genomics/informatics resource to identify predictors of tacrolimus blood concentrations in kidney transplant recipients. American Journal of Transplantation 11: 273.

Cho JH, Jin MK, Yoon YD, Park JY, Song EJ, et al. (2011) Impact of CYP3A and ABCB1 polymorphisms on tacrolimus dose-adjusted trough concentrations among renal transplant recipients in Korea. Transplant International 24: 284.

Elens L, Van Schaik R, Panin N, De Meyer M, Wallemacq P, et al. (2011) Effect of a new functional CYP3A4 polymorphism on tacrolimus dose requirement and trough blood levels in stable renal transplant patients. Therapeutic Drug Monitoring 33: 507.

Garcia M, MacIas RM, Caravaca F, Benitez J, Cubero J, et al. (2011) Polimorphisms in the CYP3A4, CYP3A5 and abcb1 genes modify tacrolimus pharmacokinetics and pharmacodynamics in renal transplant. Basic and Clinical Pharmacology and Toxicology 109: 64.

Li JL, Wang XD, Wang CX, Huang M (2011) Influence of CYP3A4, CYP3A5, ABCB1, and NR1I2 polymorphisms on tacrolimus concentrations in Chinese renal transplant recipients in the early postoperative period. Drug Metabolism Reviews 43: 72.

Satoh S, Miura M, Kagaya H, Saito M, Numakura K, et al. (2011) Impact of the CYP3A4null1G polymorphism and its combination with CYP3A5 genotypes on tacrolimus pharmacokinetics in Japanese renal transplant recipients. American Journal of Transplantation 11: 83.

Toroslu E, Sozeri B, Mir S, Berdeli A (2011) The effectiveness or the effectiveless of CYP3A4 polymorphism on calsineurin inhibitor drugs trough levels in pediatric kidney transplantations. Pediatric Nephrology 26: 1601-1602.

Yoon SH, Jin MK, Kwon O, Hong K, Cho JH, et al. (2011) Pharmacokinetics and pharmacodynamics of tacrolimus and its metabolites in kidney transplanted patients; the relationship with the CYP3A4, CYP3A5, MDR-1 genotypes. Transplant International 24: 221.

袁洪, 荆宁宁, 黄志军, 左笑丛, 席兰艳, et al. (2011) 肾移植后高血压患者应用他克莫司血药浓度的影响因素研究. 第十三次全国心血管病学术会议. 中国黑龙江哈尔滨. pp. 2.

Ares OM, Rodriguez RG, Ruiz AT, Tagarro EF, Henriquez MIG, et al. (2010) Effect of CYP3A5null1/null3 polymorphism in Tacrolimus dose requirements in kidney transplantation. Tissue Antigens 75: 605.

De Jonge H, De Loor H, Verbeke K, Vanrenterghem Y, Kuypers DR (2010) Anti-rejection therapy with high doses of methylprednisolone induces in vivo CYP3A4/5-activity in renal transplant recipients, however with limited impact on calcineurin inhibitor pharmacokinetics. Clinical Pharmacology and Therapeutics 87: S25.

Quaglia M, Fenoglio R, Antoniotti R, Lazzarich E, Airoldi A, et al. (2010) A simple pharmacokynetic parameter identifies tacrolimus slow-metabolising transplanted patients exposed to higher risk of nephrotoxicity. NDT Plus 3: i255.

Toroslu E, Sozeri B, Mir S, Berdeli A (2010) The effectiveness or the effectiveless of CYP3A4 polymorphism on calsineurin inhibitor drugs trough levels in pediatric kidney transplantations. Pediatric Nephrology 25: 1946.

黄民 (2010) 基于PGPK及中药-药物相互作用的个体化用药在中国肾移植病人中的应用. 合理用药及新药评价专题研讨会. 中国山东烟台. pp. 1

Chandel N, Minz M, Jha V (2010) Variations in CYP3A4, CYP3A5 and MDR1 have an impact on gene transcription and activity: Importance in CNI metabolism. Nephrology 15: 70

**Paediatric(3)**

Turolo S, Tirelli AS, Ferraresso M, Ghio L, Belingheri M, et al. (2010) Frequencies and roles of CYP3A5, CYP3A4 and ABCB1 single nucleotide polymorphisms in Italian teenagers after kidney transplantation. Pharmacological Reports 62: 1159-1169.

D'Alessandro LCA, Mital S (2013) Pediatric transplantation: Opportunities for pharmacogenomics and genomics. Personalized Medicine 10: 397-404.

Froger C, Promis AS, Picard N, Saint-Marcoux F, Salas C, et al. (2013) A pharmacokinetic interaction between tacrolimus and nicardipine in a pediatric renal transplant with cytochrome P450 3A loss-of-function genotypes. Fundamental and Clinical Pharmacology 27: 71.

**Pure pharmacokinetic study(4)**

Wang P, Mao Y, Razo J, Zhou X, Wong STC, et al. (2010) Using genetic and clinical factors to predict tacrolimus dose in renal transplant recipients. Pharmacogenomics 11: 1389-1402.

Ogasawara K, Chitnis SD, Gohh RY, Christians U, Akhlaghi F (2013) Multidrug resistance-associated protein 2 (MRP2/ABCC2) haplotypes significantly affect the pharmacokinetics of tacrolimus in kidney transplant recipients. Clinical Pharmacokinetics 52: 751-762.

Zuo XC, Ng CM, Barrett JS, Luo AJ, Zhang BK, et al. (2013) Effects of CYP3A4 and CYP3A5 polymorphisms on tacrolimus pharmacokinetics in Chinese adult renal transplant recipients: A population pharmacokinetic analysis. Pharmacogenetics and Genomics 23: 251-261.

Passey C, Birnbaum AK, Brundage RC, Oetting WS, Israni AK, et al. (2011) Dosing equation for tacrolimus using genetic variants and clinical factors. British Journal of Clinical Pharmacology 72: 948-957

**Healthy subjects(3)**

Jiao Z, Shi X, Geng F, Cui X, Qiu X, et al. (2010) The influence of CYP3A4 and CYP3A5 genotype and phenotype on the pharmacokinetics of tacrolimus in healthy Chinese. Clinical Pharmacology and Therapeutics 87: S52.

Choi JH, Lee YJ, Jang SB, Lee JE, Kim KH, et al. (2007) Influence of the CYP3A5 and MDR1 genetic polymorphisms on the pharmacokinetics of tacrolimus in healthy Korean subjects. British Journal of Clinical Pharmacology 64: 185-191.

施孝金, 李中东, 钟明康, 余琛, 李水军 (2010) 他克莫司在中国健康人体内的药动学特征. 中国医药工业杂志 41: 434-437

**Not primary studies(5)**

De Jonge H, Kuypers DR (2013) Response to CYP3A5 genotype, but not CYP3A4*1b, CYP3A4*22, or hematocrit, predicts tacrolimus dose requirements in Brazilian renal transplant patients. Clinical Pharmacology and Therapeutics 94: 202-203.

Santoro AB, Struchiner CJ, Felipe CR, Tedesco-Silva H, Medina-Pestana JO, et al. (2013) CYP3A5 genotype, but not CYP3A4*1b, CYP3A4*22, or hematocrit, predicts tacrolimus dose requirements in Brazilian renal transplant patients. Clinical Pharmacology and Therapeutics 94: 201-202.

Ten Brink MH, Van Der Straaten T, Bouwsma H, Baak-Pablo R, Guchelaar HJ, et al. (2013) Pharmacogenetics in transplant patients: Mind the mix. Clinical Pharmacology and Therapeutics 94: 443-444.

26. (2008) Tacrolimus dose adjustment according to CYP3A5 genotype. Pharmacogenomics 9: 1586-1587.

27. (2008) CYP3A5 as a genetic determinant of the clinical variability of CYP3A-mediated drug interactions involving tacrolimus. Pharmacogenomics 9: 1587

**Irrelevant to CYP3A4*1B and tacrolimus(39)**

Elens L, Hesselink DA, Bouamar R, Budde K, De Fijter JW, et al. (2014) Impact of POR*28 on the pharmacokinetics of tacrolimus and cyclosporine A in renal transplant patients. Therapeutic Drug Monitoring 36: 71-79.

Li CJ, Li L, Lin L, Jiang HX, Zhong ZY, et al. (2014) Impact of the CYP3A5, CYP3A4, COMT, IL-10 and POR Genetic Polymorphisms on Tacrolimus Metabolism in Chinese Renal Transplant Recipients. PLoS One 9: e86206.

Elens L, Capron A, Van Schaik RH, De Meyer M, De Pauw L, et al. (2013) Impact of CYP3A4*22 allele on tacrolimus pharmacokinetics in early period after renal transplantation: Toward updated genotype-based dosage guidelines. Therapeutic Drug Monitoring 35: 608-616.

Van Schaik R, Laure E, Dennis H, Vincent H, Ron M, et al. (2013) CYP3A4null22: A pharmacogenetic marker for CYP3A4 activity. Drug Metabolism and Drug Interactions 28: A45.

Li DY, Chen YH, Fang Y (2013) CYP3A4/5 haplotype will affect serum tacrolimus trough blood level. Journal of International Pharmaceutical Research 40: 350-354.

Li DY, Teng RC, Zhu HJ, Fang Y (2013) CYP3A4/5 polymorphisms affect the blood level of cyclosporine and tacrolimus in Chinese renal transplant recipients. International Journal of Clinical Pharmacology and Therapeutics 51: 466-474.

Yoon SH, Cho JH, Kwon O, Choi JY, Park SH, et al. (2013) CYP3A and ABCB1 genetic polymorphisms on the pharmacokinetics and pharmacodynamics of tacrolimus and its metabolites (M-I and M-III). Transplantation 95: 828-834.

Boso V, Herrero MJ, Bea S, Galiana M, Marrero P, et al. (2013) Increased hospital stay and allograft disfunction in renal transplant recipients with Cyp2c19 AA variant in SNP rs4244285. Drug Metabolism and Disposition 41: 480-487.

何霞, 童荣生 (2013) CYP3A4和CYP3A5基因多态性对汉族肾移植患者他克莫司血药浓度的影响. 中国药师 16: 497-501.

李丹滢, 陈燕华, 方芸 (2013) CYP3A4/5单倍型影响他克莫司血药谷浓度. 国际药学研究杂志 40: 350-354

Zhu L, Song HT, Wang QH, Wu WZ, Yang SL, et al. (2012) Effect of CYP3A4*18B, CYP3A5*3 gene polymorphism on dosage and concentration of tacrolimus in renal transplant patients. Yaoxue Xuebao 47: 878-883.

李丹滢, 方芸 (2010) CYP3A4*18B基因多态性与他克莫司血药浓度的相关性研究. 中国药理学会药学监护专业委员会第二届第二次国际学术研讨会. 中国江苏南京. pp. 1.

Van Schaik RH, Elens L, Bouamar R, Hesselink DA, Haufroid V, et al. (2012) The new cyp3a4 intron 6 polymorphism (CYP3A4null22) is significantly associated with decreased tacrolimus metabolism. Clinical Pharmacology and Therapeutics 91: S107.

Cho JH, Yoon YD, Park JY, Song EJ, Choi JY, et al. (2012) Impact of cytochrome P450 3A and ATP-binding cassette subfamily B member 1 polymorphisms on tacrolimus dose-adjusted trough concentrations among Korean renal transplant recipients. Transplantation Proceedings 44: 109-114.

朱琳, 宋洪涛, 王庆华, 吴卫真, 杨顺良, et al. (2012) CYP3A4*18B和CYP3A5*3基因多态性对肾移植患者他克莫司剂量及浓度的影响. 药学学报 47: 878-883.

林玲, 宋文利, 沈中阳, 张弋 (2012) 肾移植受者CYP3A5＊3和CYP3A4＊18B对他克莫司药动学的影响. 中华器官移植杂志 33: 220-224.

李丹滢, 方芸 (2012) CYP3A4*18B基因多态性与他克莫司血药浓度的相关性研究. 药学与临床研究 20: 12-15

Elens L, Bouamar R, Hesselink DA, Haufroid V, Van Der Heiden IP, et al. (2011) A new functional CYP3A4 intron 6 polymorphism significantly affects tacrolimus pharmacokinetics in kidney transplant recipients. Clinical Chemistry 57: 1574-1583.

Elens L, Van Schaik RH, Panin N, De Meyer M, Wallemacq P, et al. (2011) Effect of a new functional李丹滢, 方芸 (2010) CYP3A4*18B基因多态性与他克莫司血药浓度的相关性研究. 中国药理学会药学监护专业委员会第二届第二次国际学术研讨会. 中国江苏南京. pp. 1.

CYP3A4 polymorphism on calcineurin inhibitorsa(euro)(trademark) dose requirements and trough blood levels in stable renal transplant patients. Pharmacogenomics 12: 1383-1396.

Miura M, Satoh S, Kagaya H, Saito M, Numakura K, et al. (2011) Impact of the CYP3A4*1G polymorphism and its combination with CYP3A5 genotypes on tacrolimus pharmacokinetics in renal transplant patients. Pharmacogenomics 12: 977-984.

Ashavaid T, Raje H, Shalia K, Shah B (2010) Effect of gene polymorphisms on the levels of calcineurin inhibitors in Indian renal transplant recipients. Indian Journal of Nephrology 20: 146-151.

侯明明, 侯颖, 宋洪涛, 王庆华, 杨顺良, et al. (2010) 肾移植患者CYP3A4基因多态性对他克莫司疗效和不良反应的影响. 中国药房: 2427-2429.

Bandur S, Petrasek J, Hribova P, Novotna E, Brabcova I, et al. (2008) Haplotypic structure of abcb1/mdr1 gene modifies the risk of the acute allograft rejection in renal transplant recipients. Transplantation 86: 1206-1213

Werk AN, Lefeldt S, Bruchmueller H, Hemmrich-Stanisak G, Franke A, et al. (2014) Identification and characterization of a novel CYP3A4 variant resulting from missense mutation found in kidney transplanted patient with defects in tacrolimus clearance. Drug Metabolism Reviews 45: 76.

Werk AN, Lefeldt S, Bruckmueller H, Hemmrich-Stanisak G, Franke A, et al. (2013) Identification and Characterization of a Defective CYP3A4 Genotype in a Kidney Transplant Patient With Severely Diminished Tacrolimus Clearance. Clinical Pharmacology and Therapeutics.

Elens L, Hesselink DA, van Schaik RHN, van Gelder T (2013) The CYP3A4*22 allele affects the predictive value of a pharmacogenetic algorithm predicting tacrolimus predose concentrations. British Journal of Clinical Pharmacology 75: 1545-1547.

李丹滢, 方芸 (2012) CYP3A4~*18B基因多态性与他克莫司血药浓度的相关性研究. 药学与临床研究: 12-15.

De Jonge H, Metalidis C, Naesens M, Lambrechts D, Kuypers DRJ (2011) The P450 oxidoreductase *28 SNP is associated with low initial tacrolimus exposure and increased dose requirements in CYP3A5-expressing renal recipients. Pharmacogenomics 12: 1281-1291.

李丹滢, 方芸 (2010) CYP3A4*18B基因多态性与他克莫司血药浓度的相关性研究. 中国药理学会药学监护专业委员会第二届第二次国际学术研讨会. 中国江苏南京. pp. 1.

侯明明, 宋洪涛, 王庆华, 杨顺良, 谭建明. 肾移植患者CYP3A4基因多态性对他克莫司血药浓度/剂量比及疗效的影响. 福建省科协第九届学术年会卫星会议——福建省药学会2009年学术年会论文集; 2009 2009-01-01; 福州. pp. 7-11.

Loh PT, Lou HX, Zhao Y, Chin YM, Vathsala A (2008) Significant Impact of Gene Polymorphisms on Tacrolimus But Not Cyclosporine Dosing in Asian Renal Transplant Recipients. Transplantation Proceedings 40: 1690-1695.

Mourad M, Wallemacq P, De Meyer M, Brandt D, Van Kerkhove V, et al. (2006) The influence of genetic polymorphisms of cytochrome P450 3A5 and ABCB1 on starting dose- and weight-standardized tacrolimus trough concentrations after kidney transplantation in relation to renal function. Clinical Chemistry and Laboratory Medicine 44: 1192-1198.

Op Den Buijsch RAM, Cheung CY, De Vries JE, Wijnen PAHM, Van Dieijen-Visser MP, et al. (2006) Influence of cytochrome P450 3A5 and multidrug resistance-1 gene single nucleotide polymorphisms (SNPs) on the tacrolimus area under the curve (AUC) in renal transplant recipients. Nederlands Tijdschrift voor Klinische Chemie en Laboratoriumgeneeskunde 31: 204-206

聂新民, 黄祖发, 桂嵘, 赵红珊, 叶启发, et al. (2005) 肾移植患者血他克莫司浓度/剂量比与CYP3A5基因多态性的关系. 中华器官移植杂志 26: 58-59.

张鑫, 刘志红, 郑敬民, 陈朝红, 唐政, et al. (2004) 细胞色素P450 3A5和多药耐药基因1基因多态性在肾移植患者他克莫司血药浓度监测中的应用. 肾脏病与透析肾移植杂志 13: 313-317.

Jun KR, Lee W, Jang MS, Chun S, Song GW, et al. (2009) Tacrolimus concentrations in relation to CYP3A and ABCB1 polymorphisms among solid organ transplant recipients in Korea. Transplantation 87: 1225-1231.

Kuypers DRJ, de Jonge H, Naesens M, Vanrenterghem Y (2010) A prospective, open-label, observational clinical cohort study of the association between delayed renal allograft function, tacrolimus exposure, and CYP3A5 genotype in adult recipients. Clinical Therapeutics 32: 2012-2023.

Kuypers DRJ, Naesens M, De Jonge H, Lerut E, Verbeke K, et al. (2010) Tacrolimus dose requirements and CYP3A5 genotype and the development of calcineurin inhibitor-associated nephrotoxicity in renal allograft recipients. Therapeutic Drug Monitoring 32: 394-404

**No explicit post-transplantation time(2)**

Chitnis SD, Ogasawara K, Schniedewind B, Gohh RY, Christians U, et al. (2013) Concentration of tacrolimus and major metabolites in kidney transplant recipients as a function of diabetes mellitus and cytochrome P450 3A gene polymorphism. Xenobiotica 43: 641-649.

Op Den Buijsch RAM, Christiaans MHL, Stolk LML, De Vries JE, Cheung CY, et al. (2007) Tacrolimus pharmacokinetics and pharmacogenetics: Influence of adenosine triphosphate-binding cassette B1 (ABCB1) and cytochrome (CYP) 3A polymorphisms. Fundamental and Clinical Pharmacology 21: 427-435.

**No variant in the study population** (1)

Chakkera HA, Chang YH, Bodner JK, Behmen S, Heilman RL, et al. (2013) Genetic differences in Native Americans and tacrolimus dosing after kidney transplantation. Transplantation Proceedings 45: 137-141.

**Data unavailable** (3)

De Jonge H, De Loor H, Verbeke K, Vanrenterghem Y, Kuypers DRJ (2013) Impact of CYP3A5 genotype on tacrolimus versus midazolam clearance in renal transplant recipients: New insights in CYP3A5-mediated drug metabolism. Pharmacogenomics 14: 1467-1480.

Kuypers DR, De Jonge H, Naesens M, Vanrenterghem Y (2008) Effects of CYP3A5 and MDR1 single nucleotide polymorphisms on drug interactions between tacrolimus and fluconazole in renal allograft recipients. Pharmacogenetics and Genomics 18: 861-868.

Roy JN, Barama A, Poirier C, Vinet B, Roger M (2006) Cyp3A4, Cyp3A5, and MDR-1 genetic influences on tacrolimus pharmacokinetics in renal transplant recipients. Pharmacogenetics and Genomics 16: 659-665.
