# Supplementary material for: Effects of the CYP3A4*1B Genetic Polymorphism on the Pharmacokinetics of Tacrolimus in Adult Renal Transplant Recipients: A Meta-Analysis
Source: PLoS One. 2015 Jun 3;10(6):e0127995. doi: 10.1371/journal.pone.0127995 (PMC4454552; doi:10.1371/journal.pone.0127995)
Supplement: S2 File — (DOC) [file pone.0127995.s004.doc]

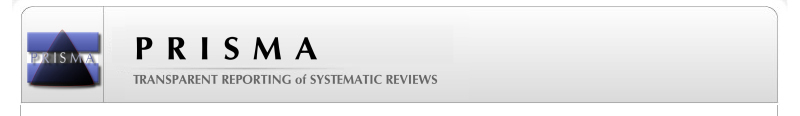
**PRISMA 2009 Flow Diagram**

**Screening**

**Included**

**Eligibility**

**Identification**

Records identified through database searching
(n = 916 )

Additional records identified through other sources
(n = 0 )

Records after duplicates removed
(n = 683 )

Records screened
(n = 683 )

Records excluded
(n = 590 )

Full-text articles assessed for eligibility
(n = 93 )

Full-text articles excluded, with reasons
(n = 80 )

Studies included in qualitative synthesis
(n = 13 )

Studies included in quantitative synthesis (meta-analysis)
(n = 7 )
